# A Two-Visit Pulpotomy Approach for the Management of Uncontrolled Bleeding in a Crown-Root Fractured Immature Incisor: A Case Report

**DOI:** 10.3390/dj14050275

**Published:** 2026-05-07

**Authors:** Holger Jungbluth, Søren Jepsen, Claudia Yvonne Schneider

**Affiliations:** Department of Operative Dentistry and Periodontology, University Hospital, Faculty of Medicine, University of Bonn, Welschnonnenstr. 17, 53111 Bonn, Germany; soeren.jepsen@ukbonn.de (S.J.);

**Keywords:** endodontics, pulpotomy, hemorrhage, hemostasis, tooth injuries

## Abstract

**Background/Objective**: The present case demonstrates the successful preservation of a severely inflamed dental pulp despite uncontrolled bleeding occurring during pulpotomy. This novel therapeutic approach was developed when a 7-year-old boy presented for endodontic treatment one week after sustaining a crown-root fracture in an immature incisor. The tooth exhibited slight hypersensitivity to cold testing, with no tenderness to percussion or palpation. An exposed pulp was present; however, when he could avoid contact with the exposed pulp tissue, the patient reported no symptoms. **Methods**: The critical determinant of success when uncontrolled pulpal bleeding occurred following pulpotomy was the stepwise treatment in two visits and the bacteria-tight seal of the access cavity. The seal was achieved through the incremental application of the temporary filling material. A calcium hydroxide preparation was applied as interappointment topical pulp dressing. Four weeks after the initial visit the pulpotomy could be accomplished. Results: During 3.5 years of follow-up, the patient remained without symptoms and the tooth showed ongoing root growth in length and width combined with positive reaction to electric pulp testing. **Conclusions**: In summary, this case demonstrates that, despite persistent bleeding following pulpotomy, pulpal vitality can be preserved, thereby promoting continued root development.

## 1. Introduction

Vital pulp therapy (VPT) has been successfully employed for decades in pediatric dentistry and dental traumatology, particularly in immature permanent teeth with pulp exposure, where preservation of pulpal vitality is essential for continued root development and long-term tooth survival [1,2,3,4]. The principal procedures encompassed by VPT include indirect and direct pulp capping, partial pulpotomy, and full pulpotomy. Carious exposures and dental trauma constitute typical indications. Even when considered alone, dental trauma accounts for a substantial portion of required treatments. The most common indications for VPT in cases of dental trauma are complicated crown fractures (CCFs) and complicated crown-root fractures (CCRFs). CCFs and CCRFs are reported in 2.6–7.1% and 0.9–4.6% of trauma cases, respectively [5,6,7,8,9], and occur most frequently in children aged 6–12 years [6,10,11,12], often necessitating endodontic intervention.

In such cases, the classical approach is partial pulpotomy according to Cvek, in which a limited portion of the coronal pulp is removed and covered with a biocompatible material [13]. Reported success rates exceed 96% [3,13,14]. However, when treatment initiation is delayed for any reason and pulpitis has to be presumed, a central challenge remains the lack of reliable diagnostic tools for assessing the true extent of pulpal inflammation [15]. At present, intraoperative bleeding control following pulpotomy serves as the key clinical criterion. Current recommendations propose that hemostasis should be achieved within about 5 min using sodium hypochlorite irrigation (NaOCl); failure to do so would necessitate stepwise progression from partial to full pulpotomy, and ultimately to pulpectomy if bleeding persists [16,17,18,19]. However, when complete pulpectomy and subsequent apexification become necessary, the stage of root development becomes a decisive prognostic factor: the less mature the root, the higher the risk of cervical root fracture following treatment [20,21]. While earlier studies on immature teeth with pulpal necrosis primarily relied on long-term calcium hydroxide dressings, current practice favors apexification procedures using hydraulic calcium silicate cements (HCSCs). Nevertheless, it remains evident that the more immature a root, the lower its mechanical stability, and the more critical successful VPT becomes in enabling continued root development.

Thus, in teeth with very immature roots and uncontrolled pulpal bleeding, clinicians face a therapeutic dilemma: further amputation of pulp tissue may allow immediate preservation of the tooth, but the long-term prognosis remains poor. Without continued root maturation, mechanical stability is compromised, leaving the tooth at high risk of fracture.

This case report illustrates the successful VPT of an immature incisor with a CCRF and severely inflamed pulp, despite delayed treatment initiation and uncontrolled bleeding during pulpotomy.

## 2. Case Report and Clinical Techniques

A seven-year-old systemically healthy boy presented in the Department of Periodontology, Operative and Preventive Dentistry at the Centre for Dental and Oral Medicine of the University Bonn seven days after dental trauma (Figure 1). The extraoral findings were unremarkable.

Primary care was taken in the emergency service of the maxillofacial surgery department on the day of injury. The injury comprised avulsion of teeth 21 and 22 and a CCRF of tooth 11. While tooth 21 had been replanted and splinted with a flexible trauma splint, tooth 22 was lost and could not be found anymore (Figure 2). The pulpal exposure of tooth 11 had been covered with a temporary calcium hydroxide liner, which had since been lost.

Tooth 11 showed a slightly increased response to cold thermal stimulation (Kältespray classic, Omnident Dental-GmbH, Rodgau, Germany), while electric pulp testing (EPT) (Digitest^®^ II, Parkell, Edgewood, NY, USA) yielded 11/64. The tooth exhibited no tenderness to percussion or palpation and demonstrated physiologic mobility. The patient reported no complaints as long as he avoided contact with the re-exposed pulpal tissue.

Periodontal probing and a radiograph revealed, that the hard tissue defect of the fracture extended to a deep subgingival and even subcrestal level of about 3 to 4 mm (red arrow in Figure 3a). The tooth was not yet fully erupted and root development was incomplete with thin dentinal walls and a wide-open apex (Figure 3a). All radiographs were obtained using a KaVo InExam tube (KaVo Dental GmbH, Biberach an der Riß, Germany) and a Xios XG sensor size 2 (Dentsply Sirona GmbH, Bensheim, Germany) at 60 kV, 4 mA, with an exposure time of 0.16 s.

Therefore, the diagnosis for tooth 11 was CCRF with asymptomatic irreversible pulpitis and normal apical tissues [23]. Although the delay in treatment initiation indicated possible complications, partial pulpotomy was intended in order to maintain pulp vitality and allow further root development. Informed, valid consent was obtained from the patient and his parents. After administration of local anesthesia using articaine with epinephrine (Ubistesin^TM^, 3M ESPE, Seefeld, Germany), the splint was removed from teeth 12 and 11, and two composite attachments were adhered on the vestibular and palatal surfaces of tooth 11 to allow rubber dam application and retention with a clamp.

The tooth was carefully cleaned and disinfected with 1% NaOCl and about 2 mm of pulpal tissue was removed with a sterile diamond bur under water cooling. It was attempted to control the resulting pulpal bleeding with cautious irrigation using NaOCl and sterile paper points, but the bleeding could not be stopped. After another 5 min of trial, further pulpal tissue was removed attempting to reach hemostasis. However, even after stepwise tissue removal extended to the cervical third of the root, hemostasis could not be achieved. The pulp consistently appeared intact and vital, without evidence of degeneration, already after removal of the initial 2 mm. Only the persistent bleeding complicated the planned treatment. Pulp tissue removal was, therefore, stopped at this position and again it was attempted to control the continuing bleeding. Alternating NaOCl rinsing and drying with sterile paper points of gross diameter were employed for about 40 min, which is considerably longer than typically recommended or considered clinically acceptable. However, the bleeding still persisted and thus, with the consent of the patient and his parents, the strategy was changed at this point. Instead of completing pulpotomy in one visit with a definitive capping of the remaining pulpal tissue, a temporary capping was now intended. Another appointment was planned to be scheduled to give the pulp a chance to recover in the meantime and to enable proper hemostasis and permanent application of a definitive capping material at the second visit.

A non-setting calcium hydroxide paste (Calxyl^®^, OCO Präparate GmbH, Dirmstein, Germany) was therefore placed onto the continuing bleeding as well as possible and the access cavity was temporarily sealed by incremental application of a temporary filling material (CavitTM, 3M ESPE, Seefeld, Germany). It was thereby achieved to contain the blood flow below the temporary filling, and a coverage layer of bonded flowable composite was applied on top to ensure an adequate seal against the oral cavity. The splint was reattached.

Two weeks later, the patient returned without any symptoms with respect to tooth 11. There was no tenderness to percussion or palpation; however, the tooth exhibited no response to cold testing or EPT (64/64) at this appointment. A radiograph was taken, revealing no signs of inflammatory reaction or resorption at the apical part of the root (Figure 3b). In accordance with the patient another appointment was scheduled another two weeks later to re-enter the access cavity and complete pulpotomy by definitive pulp capping and sealing of the cavity.

At the four-week follow-up visit after incomplete pulpotomy, the tooth was non-responsive to cold testing but responded to EPT (32/64); the patient remained asymptomatic. After definitive removal of the splint, local anesthesia was administered. The tooth was isolated with rubber dam, the surfaces were disinfected with NaOCl and the temporary sealing was removed. Only minimal bleeding occurred when the position was reached, where previous treatment had been halted. The bleeding was easily controlled by rinsing with NaOCl and paper points. A fragile hard tissue barrier was observed at this level under a dental operating microscope. The barrier was left in place and carefully covered with calcium hydroxide (Calxyl^®^, OCO Präparate GmbH, Dirmstein, Germany). The calcium hydroxide paste was covered with a thin layer of light curing glass ionomer cement (VitreBondTMPlus, 3M, Seefeld, Germany), and the access cavity was then definitely sealed with composite. A postoperative radiograph was taken. No signs of apical pathology were present and the cervical periodontal tissues at the mesial aspect appeared to reorganize spontaneously (Figure 3c).

A mesial composite build-up only of the supragingival defect was placed without any manipulation of the subgingival defect in order to avoid impairment of the periodontal healing. The procedure was performed under rubber dam isolation, using a standard adhesive (Optibond^TM^ FL, Kerr, Orange, CA, USA) and composite material (Tetric^®^ EvoCeram & EvoFlow, Ivoclar Vivadent, Schaan, Liechtenstein) with a custom-fitted transparent matrix.

Clinical follow-ups were regularly done after three, six, twelve, twenty-four (2 y), and 42 months (3.5 y). In the meantime, root canal treatment and MTA apexification was performed on tooth 21 due to development of a pulp necrosis with inflammatory root resorption. Figure 4 illustrates the clinical presentation of tooth 11, demonstrating satisfactory aesthetics and healthy periodontal conditions.

Tooth 11 remained asymptomatic with clinical and radiographic signs of healing (Figure 3d–f). It showed positive reaction to EPT at the 3-month follow-up (54/64) and during the whole recall period (6 mo: 34/64; 12 mo: 37/64; 24 mo: 25/64; 42 mo: 27/64), but no reaction to the cold test could be elicited. Periodontal probing depths were within normal limits, even at the mesial aspect, where the fracture initially extended to the subcrestal level. Follow-up radiographs at 12 and 24 months post-treatment (Figure 3d,e) reveal progressive root maturation, characterized by increasing root wall thickness and elongation. At 42 months, radiographic evidence of pulp canal obliteration was observed (red arrow in Figure 3f). At follow-up, the patient, now 11 years old, reported, as assessed by use of an age-appropriate visual analog scale, that while the treatment had been moderately burdensome in retrospect, he currently experienced optimal oral function, no limitations in daily activities, and expressed high satisfaction with the therapeutic outcome. A 4-year clinical follow-up evaluation has been scheduled.

## 3. Discussion

The present case highlights that, even in the presence of severe pulpal inflammation and uncontrolled bleeding VPT can achieve successful preservation of radicular pulp tissue and promote continued root development in an immature permanent incisor despite delayed treatment initiation. To the best of our knowledge, this is the first report of a case of this kind. A two-visit approach was employed to manage uncontrolled bleeding following full pulpotomy, aiming to avoid further pulp tissue removal as hemostasis could not be achieved at the first appointment.

Adequate hemostasis is prerequisite for the successful execution of all forms of VPT. Due to the lack of alternative diagnostic methods, hemostasis serves as the “state of the art” confirmation to the clinician of having reached healthy remaining pulp tissue [19]. If hemostasis cannot be achieved after approximately 5 min, most authors and position statements recommend further pulp tissue removal in order to reach an underlying area where the tissue is non-inflamed and bleeding can be controlled [16,17,18,19]. When pulpal inflammation is severe and reaching into deeper regions, this approach leads to pulpectomy and subsequent apexification or conventional root canal treatment. In teeth with advanced root formation or even mature roots the concept appears effective and practical. In cases with very immature roots, however, continued removal of pulpal tissue into the root due to persistent bleeding may impede the goals of VPT, i.e., the desired increase in root canal wall stability. This is because physiological thickening of the root canal walls can only occur apically to the level of pulp tissue amputation. Coronally to this level, the dentin walls of the crown and root remain unchanged in thickness, hindering biological stabilization of these areas.

One may hypothesize that a specific, yet unidentified, anatomical limit exists along the longitudinal axis of an immature tooth, beyond which further amputation of pulpal tissue may markedly compromise long-term biomechanical stability, particularly increasing the risk of cervical root fractures. Although this limit is not precisely defined, it can be hypothesized to be situated approximately at the level of the cemento-enamel junction (CEJ). Further research would be required to elucidate this assumption. In the present case, we aimed however not to surpass the CEJ or the extent of the fracture on the mesial root surface too far during pulpal tissue removal. Nevertheless, assessment of the radiographs indicates that at least the CEJ was exceeded. Retrospectively, it may have been more advantageous to cease pulp tissue removal earlier, even in the presence of persistent bleeding, and consider the two-visit pulpotomy approach sooner. This might have facilitated root thickening even in the more coronally situated areas, potentially extending into the cervical aspect of the crown.

In consideration of this, it should be emphasized that the bacteria-tight seal appears of utmost importance during the time between the two appointments. This can be inferred from findings regarding factors influencing the success of classical VPT and the management of deep caries lesions [15,24,25,26]. We are convinced that without a tight provisional seal, the outcome would not have been as favorable. To ensure a bacteria-tight seal in spite of persistent bleeding, the temporary filling material was introduced using an incremental technique. According to this technique, after the application of the calcium hydroxide material, at least three increments are sequentially placed. A sufficient depth of the access cavity is essential for this purpose. Such a depth may be limited by the course of fracture lines, as in the present case. The first increment is then pressed laterally against the wall of the access cavity, leaving a small area of the pulp exposed. This allows the continuous bleeding to drain without displacement of the first increment. If necessary, the interim bleeding is briefly rinsed with NaOCl before application of the second increment. The second increment then forms the actual closure of the pulp tissue to the oral cavity. Due to the persistent bleeding, it may not be possible to achieve a completely edge-tight placement, as blood may seep between the material and the wall of the access cavity. Only the third increment should be introduced in such a way that it is anchored circularly on clean walls of the access cavity. Additional increments can be introduced if needed. In the present case, to ensure a reliable bacteria-tight seal, a layer of adhesively bonded flowable composite was additionally applied.

As a temporary pulp capping material, a pure aqueous calcium hydroxide paste was used, which is also indicated for definitive pulp capping procedures. This preparation contains no additives or setting components, which may facilitate clinical handling but can compromise the biological performance of the material [27]. At the second appointment, the same material was newly applied as the definitive pulp capping agent before the access cavity was permanently restored. Although a pure calcium hydroxide paste remains acceptable as a definitive pulp capping material according to the 2021 ESE position statement on endodontic management of traumatized permanent teeth [28], the application of a non-discoloring HCSC cement might have represented a slightly more favorable alternative in this case.

At the time of the patient’s initial presentation, only a conventional radiograph was obtained despite the presence of a CCRF. Probing of the mesial fracture margin revealed a smooth, gradual transition of the fractured surface. Accordingly, the described therapeutic approach was pursued. Had there been suspicion of an additional root fracture—for example, based on detection of a sharp edge at the apical extent of the fracture surface—or to definitively exclude such a condition, cone-beam computed tomography (CBCT) would have been useful for visualization. Although the favorable clinical course suggests the absence of additional fracture lines within the root, their presence, if undetected, could have significantly compromised the prognosis of the tooth.

Severe pulpal inflammation and the inability to control bleeding at the initiation of endodontic therapy were most likely due to the patient’s delayed initial presentation to our department one week after receiving primary care on the day of injury. The delay can be attributed to a misjudgment by the parents regarding the severity of the situation, despite clear explanations provided by the maxillofacial surgeon. However, postponement of endodontic treatment may also arise from other factors. Severe multiple traumatic injuries requiring intensive care admission, delayed recognition of pulp exposure during initial management, patient non-compliance, or other variables are typical contributors to such delays. Advanced pulpal inflammation with challenging hemostasis or even partial or full pulpal necrosis may therefore result and complicate tooth preservation.

The presented two-visit pulpotomy approach was developed during treatment of the present case, arising from the lack of suitable alternatives when uncontrolled bleeding occurred, and, when at the same time, preservation of pulpal vitality and continued root growth appeared of paramount importance for biomechanical reasons. Further investigations might address the question whether an indication for this procedure can also be expanded to other cases. Cases with further-developed but not fully matured roots, where pulpal bleeding cannot be controlled during initial treatment would appear suitable candidates. In these cases, to date, the decision is routinely taken to perform pulpectomy followed by HCSC apexification. It is hoped that the presented technique could preserve pulp vitality in a number of teeth and allow further root maturation. Importantly, should the two-visit approach fail to achieve the desired outcome, conventional apexification remains a viable alternative treatment strategy (Figure 5). The two-visit approach could potentially also expand the therapeutic spectrum of VPT in mature and posterior teeth.

A research group has previously reported a similar approach to manage uncontrollable bleeding during VPT, which they called the “tampon pulpotomy” [29,30]. In this technique, a biocompatible, calcium-enriched cement, developed by the authors, is applied to the persistently bleeding pulp stump to arrest bleeding via pressure and lateral sealing. It is applied as a definitive capping during the first session. However, their reports have been limited to primary molars or permanent premolars and molars, most of which were affected by extremely deep carious lesions. The remainder of our literature search revealed no reports describing a vital pulp-preserving procedure in cases where pulpal bleeding could not be adequately controlled. Most other sources recommend further removal of pulp tissue if hemostasis cannot be achieved after 5 or 6 min [16,17,18,19,31]. In the present case, such a strategy would likely have resulted in a structurally less stable root compared with the outcome achieved.

Another recently published study describes the use of cryotherapy for hemostasis in permanent molars affected by deep or extremely deep caries and symptomatic irreversible pulpitis. At 2.5 °C in combination with saline solution, cryotherapy demonstrated a reduced time to hemostasis compared with 2.5% NaOCl [32]. The procedure was also associated with lower pain levels in the immediate post-VPT period, a finding corroborated by another investigation [33]. A 0.05% oxymetazolin-based hemostatic solution has likewise been successfully employed for hemostasis in combination with MTA during VPT in primary molars [34]. In comparison with 20% ferric sulfate and combined with a reinforced zinc oxide eugenol dental restorative material, superior outcomes were reported. However, cases in which bleeding persisted for more than six minutes were excluded from the analysis.

At the time of treatment, the authors were unaware of the “tampon pulpotomy” leading to the parallel development of a similar approach. Both methods share the principle of capping an uncontrolled pulpal bleeding with a biocompatible material and using a mechanical barrier to control bleeding. The present technique differs in that the initial material is later removed and the pulpotomy is completed in a second step. This additional intervention appears particularly important in permanent anterior teeth, given the risk of discoloration following VPT using HCSCs—especially in the presence of a blood clot. Overall, although slightly more labor-intensive, the presented technique may be better suited for anterior teeth.

However, as this report describes only one single case, no generalizable conclusions regarding prognosis can be inferred. It should also be emphasized that pulpal inflammation was asymptomatic in the present case. In addition, the possibility of risks in comparison with established treatment concepts cannot be ruled out. Even though more robust evidence from well controlled randomized trials would be desirable, this prospect does not appear to be realistic due to the enormous challenges involved. In lack of such trials, clinicians will have to rely on the information gained by properly documented case reports and/or case series.

## 4. Conclusions

Even if uncontrolled bleeding occurs during intended pulpotomy treatment, pulpal tissue could successfully be preserved in carefully selected cases, where root maturation appears of utmost importance. Division of the vital pulp treatment into two visits and achieving a bacteria-tight seal of the access cavity appear to be critical steps.

## Figures and Tables

**Figure 1:**
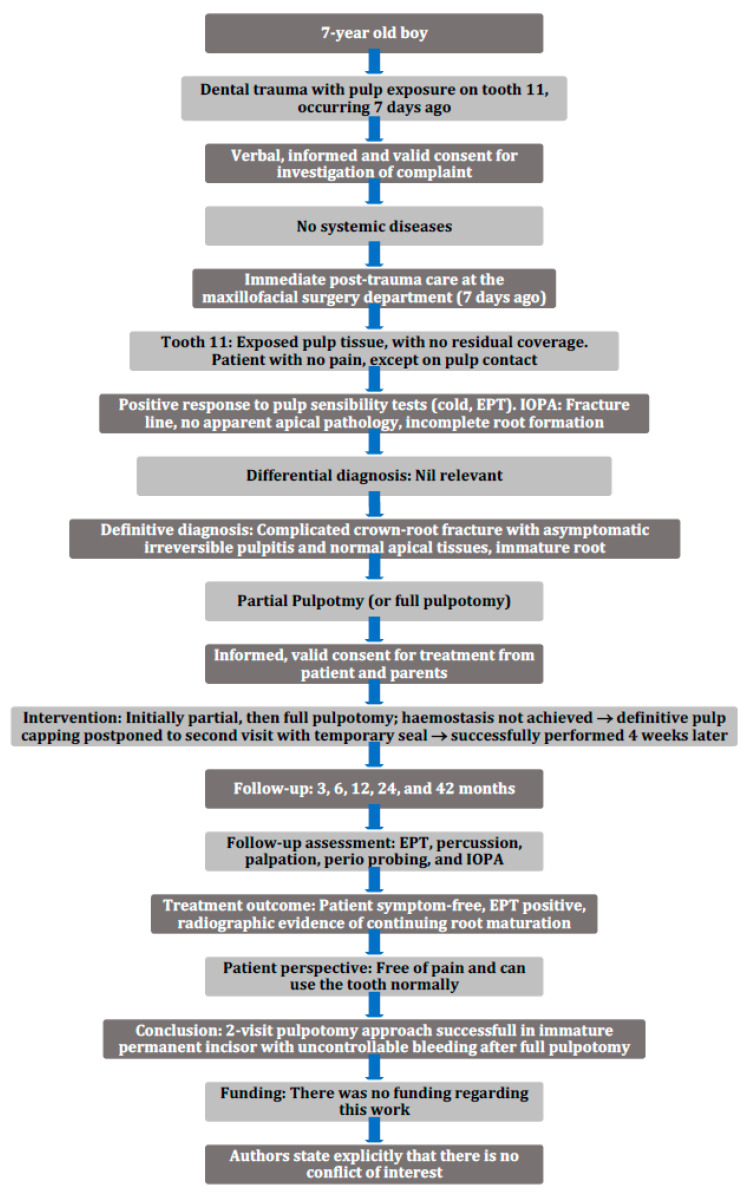
PRICE flowchart according to Nagendrababu et al. [22].

**Figure 2:**
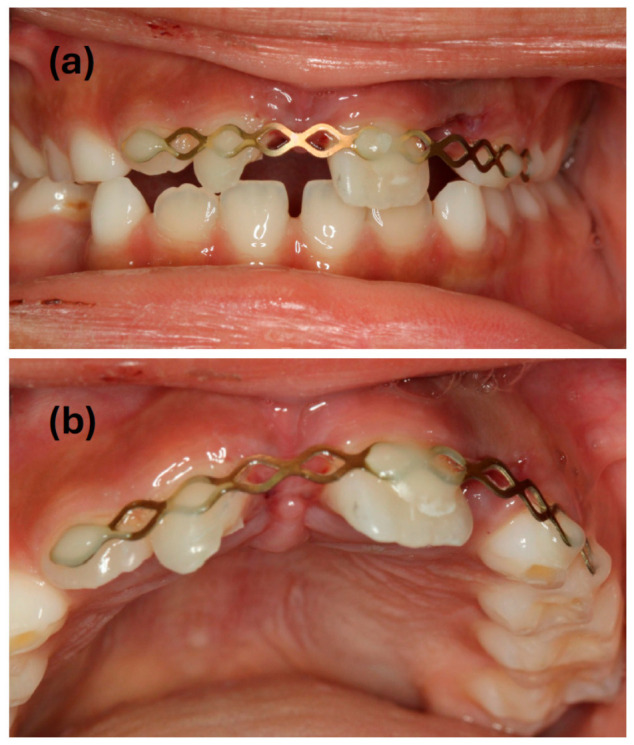
Intraoral views: (**a**) facial view and (**b**) occlusal view, following traumatic injury and immediate emergency management (7-year-old boy). Tooth 11 presents with a CCRF. Tooth 21 has been replanted and stabilized with a flexible splint. Tooth 22 is missing as a result of avulsion.

**Figure 3:**
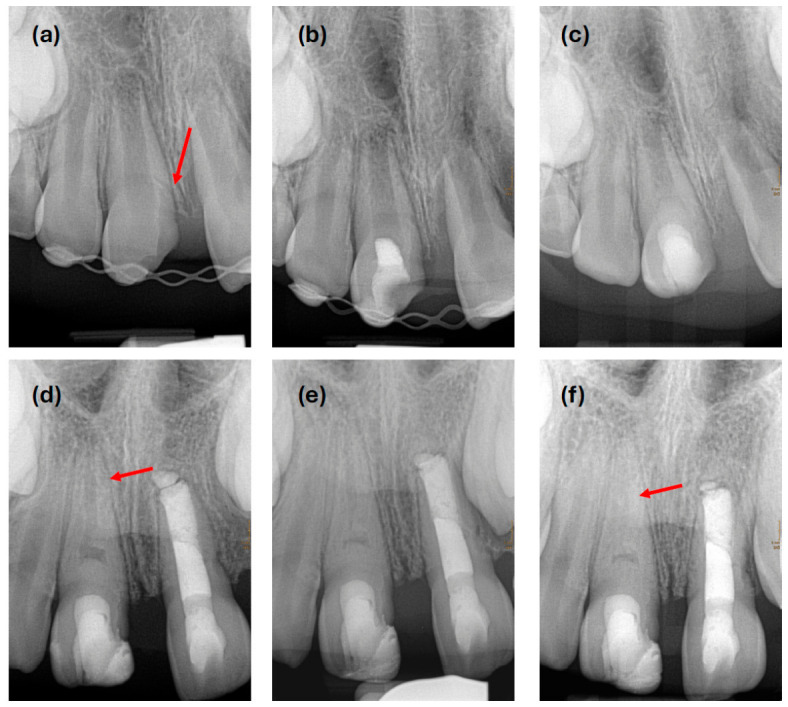
Radiographic sequence of tooth 11 after traumatic injury. (**a**) Initial presentation following emergency management at the maxillofacial department. Note the fracture line extending 3 mm subcrestally (arrow). (**b**) Follow-up radiograph two weeks after the initial pulpotomy attempt. No cervical or apical findings suggestive of progressive inflammation or resorption. The tooth remained asymptomatic. (**c**) Follow-up radiograph immediately after completed VPT. The pulpotomy extends into the cervical third of the root. (**d**) One-year follow-up radiograph. A distinct thickening and elongation of the root walls is evident (arrow). At the amputation site, a dense calcification becomes visible mesially. In the meantime, apexification of tooth 21 was required due to inflammatory root resorption. (**e**) Two-year follow-up radiograph. Continued root development with further increase in length and thickness is observed. (**f**) Finally, 3.5-year radiographic follow-up. Root formation appears complete. Apical to the amputation site, progressive calcification of the pulp space is apparent (arrow). The non-radiopaque calcium hydroxide paste appears as a radiolucent area in the cervical third of the root. A continuous physiological periodontal ligament space is observed along the mesial and distal surfaces of the root, including the area of previously subcrestal fracture line.

**Figure 4:**
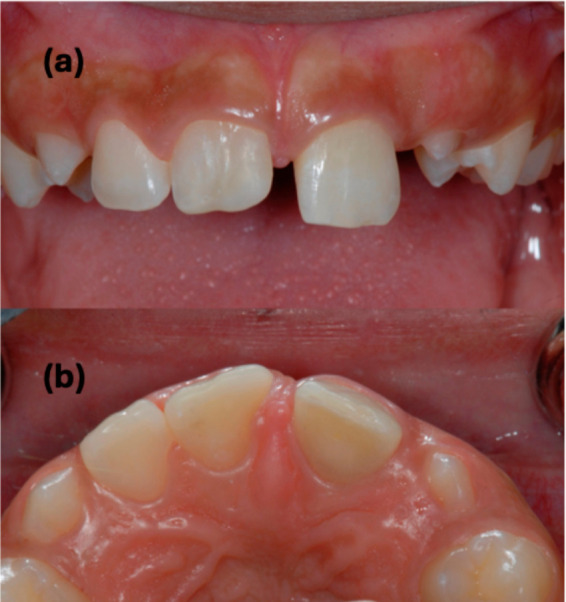
Intraoral views at 3.5-year follow-up (male patient, 11 years old). Healthy periodontal conditions are observed at tooth 11. (**a**) Facial view. (**b**) In occlusal view, tooth 21 shows slight gray discoloration following MTA apexification.

**Figure 5:**
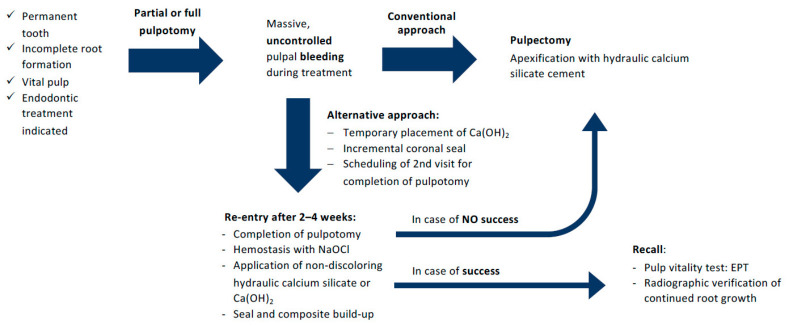
Schematic overview of the possible treatment options in the event of uncontrolled bleeding during pulpotomy of an immature tooth. If the proposed alternative approach is not successful, the conventional treatment strategy (e.g., apexification) may be pursued.

## Data Availability

All data are included in the manuscript.

## Abbreviations

The following abbreviations are used in this manuscript:

VPT	Vital Pulp Therapy
CCF	Complicated Crown Fracture
CCRF	Complicated Crown-Root Fracture
HCSC	Hydraulic Calcium Silicate Cement
EPT	Electric Pulp Test
NaOCl	Sodium Hypochlorite
MTA	Mineral Trioxid Aggregate
CEJ	Cemento-Enamel Junction
PRICE	Preferred Reporting Items for Case Reports in Endodontics
